# OCD and COVID-19: a new frontier

**DOI:** 10.1017/S1754470X20000318

**Published:** 2020-07-14

**Authors:** Amita Jassi, Khodayar Shahriyarmolki, Tracey Taylor, Lauren Peile, Fiona Challacombe, Bruce Clark, David Veale

**Affiliations:** 1 National Specialist Clinic for Young People with OCD, BDD and Related Disorders, South London and Maudsley NHS Foundation Trust, London SE5 8AZ, UK; 2 Centre for Anxiety Disorders and Trauma, South London and Maudsley NHS Foundation Trust, London SE5 8AZ, UK; 3 Institute of Psychiatry, Psychology and Neurosciences, King’s College London, London SE5 8AF, UK

**Keywords:** cognitive appraisals, cognitive behaviour therapy, obsessive compulsive disorder

## Abstract

People with obsessive compulsive disorder (OCD) are likely to be more susceptible to the mental health impact of COVID-19. This paper shares the perspectives of expert clinicians working with OCD considering how to identify OCD in the context of COVID-19, changes in the presentation, and importantly what to consider when undertaking cognitive behaviour therapy (CBT) for OCD in the current climate. The expert consensus is that although the presentation of OCD and treatment may have become more difficult, CBT should still continue remotely unless there are specific reasons for it not to, e.g. increase in risk, no access to computer, or exposure tasks or behavioural experiments cannot be undertaken. The authors highlight some of the considerations to take in CBT in light of our current understanding of COVID-19, including therapists and clients taking calculated risks when developing behavioural experiments and exposure tasks, considering viral loading and vulnerability factors. Special considerations for young people and perinatal women are discussed, as well as foreseeing what life may be like for those with OCD after the pandemic is over.

**Key learning aims:**

(1) To learn how to identify OCD in the context of COVID-19 and consider the differences between following government guidelines and OCD.
(2) To consider the presentation of OCD in context of COVID-19, with regard to cognitive and behavioural processes.
(3) Review factors to be considered when embarking on CBT for OCD during the pandemic.
(4) Considerations in CBT for OCD, including weighing up costs and benefits of behavioural experiments or exposure tasks in light of our current understanding of the risks associated with COVID-19.

## Introduction

Coronavirus disease (COVID-19) is a new and rapidly evolving pandemic. The physical consequences are well documented but mental health challenges have been less well considered (World Health Organization, 2020). Whilst COVID-19 will create problems for mental health more broadly, there are groups that are potentially more vulnerable to the impact of this pandemic; these include those with obsessive compulsive disorder (OCD).

This paper walks through the main clinical considerations when working with OCD in the context of COVID-19, including what to consider in the delivery of cognitive behaviour therapy (CBT). It is written by clinicians working with young people and adults across both in-patient and out-patient national specialist OCD services in the UK. It offers practical advice and clinical reflections, including case examples, to support others working with this client group.

## Identifying OCD in the context of COVID-19

OCD is a common disorder affecting around 1.6% of adults (Kessler *et al*., 2005) and 1–3% of children and adolescents (Heyman *et al*., 2001; Valleni-Basile *et al*., 1995). OCD can centre on recurrent or persistent obsessions or pre-occupation around a fear of contracting/suffering from an illness or fear of contamination, which drives sufferers to engage in repetitive behaviours or avoidance to reduce the risk of developing the illness. Obsessions/pre-occupation or compulsions/behaviours in these disorders are time consuming (e.g. take more than 1 hour per day) and cause clinically significant distress or impairment in social, occupational or other important areas of functioning (American Psychiatric Association, 2013; World Health Organization, 2019).

Stringent following of UK government guidelines to reduce the risk of contracting COVID-19 to an observer may mimic the presentation of some of the most publicly perceived symptoms of OCD, for example repetitive handwashing/antibacterial gel use, avoidance of potential contaminants, or socially isolating. An understandable consequence of COVID-19 is heightened anxiety due to the increased likelihood and awfulness of the threat. However, it is important to consider what the differences are between those who are following government guidelines to those with OCD to inform clinical practice. These centre on a different set of beliefs and motivations driving the behaviours, and their cessation (see Table 1).

**Table 1.** Potential differences between COVID-19 government guidelines and OCD

Covid-19 government guidelines	OCD
Wash hands or use hand gel for 20 seconds	Wash hands or use hand gel until ‘internal referenced’ criterion has been satisfied, likely to be more than 20 seconds
Wash hands or use hand gel when returning home from public place	Wash hands or use hand gel in response to an obsession, idiosyncratic trigger or pre-occupation/obsession regarding COVID-19 (likely to be frequent and excessive)
Wash hands or use hand gel generally following steps advised by government	Wash hands or use hand gel in a rigid and ritualised way
Avoid touching things others may have touched – use sanitiser	Wear gloves at all times, even at home
Do not allow anyone outside family in home	Do not allow anyone in the home
Social distance	Stop going out altogether

People without OCD are more likely to follow the guidelines in the way set out by the government (i.e. thorough handwashing for 20 seconds), whereas those with OCD may be likely to wash until meeting an ‘internally referenced criterion’ such as ‘feeling comfortable’ or ‘just right’, resulting in prolonged and repetitive behaviour that is likely to be longer than 20 seconds. They may wash their hands more frequently than those who are following COVID-19 guidance and may do so in response to an obsession (i.e. intrusive thought, doubt or sensation), an idiosyncratic trigger (e.g. after a shower) or in response to the pre-occupation with or fear that they may be at risk of contracting COVID-19, rather than a time when they may need to, such as coming home from a public place. It is likely that sufferers will clean or hand wash in a rigid or ritualised way. They may have developed methods for cleaning shopping with anti-bacterial gel, or quarantining parcels and shopping delivered for a set period of time before they can be opened. These behaviours are clearly idiosyncratic, excessive and not recommended by government guidelines. In addition to this, people with OCD may report a heightened moral responsibility for spreading COVID-19 and may engage in other behaviours such as excessive checking of COVID-19-related information and reassurance seeking. Finally, there may also be repeated consultation with medical professionals or advice lines, without lasting reassurance. The next section will consider in more detail the presentation of OCD in the context of COVID-19.

It is likely that as the current pandemic took hold and awareness increased, most individuals without OCD will have experienced some increased distress and safety-seeking behaviours. These understandable, ego-syntonic responses to an objective increase in threat should not be pathologised and, as time has passed, most of these behaviours will have reduced. Clinicians should, however, be alert for those who may start to exceed the diagnostic threshold for OCD, whose obsessions and compulsions are becoming excessive, time consuming, distressing and interfering with their lives (see Box 1 for an example). There is no evidence to suggest increased rates of OCD during or immediately after other major health scares, such as HIV, swine flu, etc. Nevertheless, if people do start to exhibit more enduring symptoms in line with OCD, this may warrant a referral for CBT (see ‘What to consider in CBT’ section below).

Box 1. Case example of OCD in context of COVID-19

A 39-year-old married man was fearful of himself and his family catching COVID-19. His children had been kept in the house for the previous 6 weeks during ‘lockdown’. He reported that parcels delivered to his home were kept in quarantine in the garden for 3 days. The garden patio would be washed down with boiled water and Dettol. All non-perishable food is sterilised by boiling in water before it can be eaten. He went out for the odd run but kept a 5 metre distance. He sprayed his door handles and bell daily with alcohol. He avoided touching taps, window locks and door handles in the house. He washed his hands about 20 times a day. His wife had to accommodate his anxiety-driven behaviours.

## OCD presentation during COVID-19

Presentations of OCD can be very idiosyncratic; some people may be relatively unscathed by the current virus, others may experience elevated and devastating degrees of distress. Some have even described that the societal restrictions in place to manage COVID-19 have led to perceived short-term benefits. For example, the ‘lockdown’ may facilitate some compulsive behaviours, such as reassurance seeking (e.g. by ensuring that household members are close by at all times) and avoidance (e.g. by reducing contact with children for those with paedophilic obsessions). Some have stated other ‘positive’ aspects such as a perceived validation of beliefs about the harmfulness of contamination, and relief and reassurance in government-mandated hygiene practices and safe distancing. Lockdown may also be preferred as it allows one to avoid the uncertainty of appropriate levels of precaution-taking.

Some sufferers have reported that the content of obsessions/pre-occupations has changed since the pandemic; those with other contamination/illness concerns are now more focused on COVID-19. Others have reported a worsening of their symptoms, which may be due to many direct and indirect factors related to COVID-19. Without doubt there is an increase in threat/risk and information about that threat; OCD sufferers are likely to have attentional biases towards threat information.

Furthermore, there is a surfeit of information from multiple sources which is rolling and instant in the digital age (Freeston *et al*., in press). Unfiltered information may be perplexing, contradictory, untrustworthy or even misinformation. The 24-hour news cycle and widely accessible information online about COVID-19 can exacerbate checking behaviours and reassurance seeking. The constant checking of information will undoubtedly create further fear and uncertainity. A worsening of symptoms can occur as people engage in a cycle of triggered excessive threat perception, anxiety and excessive compulsions, such as showering for hours after arrivals of post and food shopping, handling everything with gloves and wiping every item down. Some have reported that despite taking recommended precautions, they still ‘feel’ that COVID-19 has contaminated their homes and they experience urges to decontaminate it entirely, even to an extreme of removing wallpaper and flooring.

Intolerance of uncertainty (IOU) is a well-documented feature of OCD (Tolin *et al*., 2003; Boelen and Carleton, 2012). Various sources of uncertainty to do with the pandemic may be relevant to people with OCD. Uncertainty around whether individuals have COVID-19 or not, about the ‘right’ level of necessary precaution, receiving inconsistent or even conflicting messages from different institutions, as well as ‘the new normal’, where even people without OCD are trying to avoid potential contamination in a variety of ways, may make sufferers uncertain about whether they are taking the right approach or whether what they are doing is excessive. Additionally, with the government guidelines around washing and social distancing, some OCD sufferers are reporting difficulty in identifying which behaviours are acceptable and recommended versus what is driven by their anxiety. IOU may mainfest in a variety of responses, including repeated researching and analysing of information related to the virus and precautionary measures, reviewing whether one has carried out actions properly, or altogether avoiding tasks where the associated uncertainty is deemed unacceptably high.

Inflated responsibility, the belief ‘that one has power that is pivotal to bring about or prevent subjectively crucial negative outcomes’ which therefore obliges action (Salkovskis *et al*., 1995), is another key concept in cognitive models of OCD. When faced with the uncertainty associated with COVID-19, those with OCD may feel driven to take what they perceive as responsible action, with no upper limit to this definition. Responsibility beliefs may encompass perceptions of others as insufficiently careful, and therefore drive attempts to compensate for this by taking excessive actions to prevent harm to self or others, including checking that others have followed routines properly. For sufferers who are parents, this may extend to imposing excessive (and potentially harmful) routines on children, which may need to be factored into risk assessment. A case example of inflated responsibility is presented in Box 2.

Box 2. Case example of inflated responsibility

A young autistic woman aged 17 became distressed when her mother developed moderate symptoms of COVID-19, necessitating brief hospitalisation. She became pre-occupied with the thought that she had infected her mother (without objective evidence of this) and driven to carry out increasingly time-consuming rituals. In addition to excessive handwashing, cleaning of objects, avoidance of anything associated with her mother and frequent reassurance seeking, she also spent hours each day praying. These behaviours had been present to varying degrees previously but had responded well to CBT, so this marked a sudden deterioration. She expressed a belief that not carrying out compulsions would lead to her mother’s death and this would be entirely her fault.

Individual patterns of safety-seeking behaviour and avoidance will be meaningfully linked to cognitive themes. Those with IOU appraisals may seek absolute certainty that they have taken proper precautions, leading to repetitive actions. They may adopt subjective termination criteria for when to stop handwashing, such as feeling ‘just right’, timing or otherwise closely monitoring actions. Decision-making about how to behave may be impacted by conflicting messages about the correct levels of precaution, with the highest levels sought and adhered to. Attempts to remove contamination may become extreme, perhaps involving use of specialised products, repeated cleaning, etc. Those whose main fear is that they will spread the virus to others might engage in repeated checking (e.g. self-monitoring for possible symptoms, mentally retracing their steps and reviewing who may have had contact with), and reassurance-seeking/confessing. Extreme levels of agoraphobic avoidance may be seen as the only way to achieve certainty about one’s likelihood of causing harm and/or having acted responsibly. In summary, while the content of this threat is new, the mechanisms of response within OCD remain consistent with other well-documented stimuli.

Finally, the circumstances of lockdown including severe disruption of routine, financial and interpersonal stress and limited access to external protective factors, may lead to a vacuum that OCD can fill. Disruptions to routine and activity can contribute to low mood, which in turn increases the accessibility of intrusive thoughts and reinforces their credibility. An additional consequence of lockdown is the impact on families and partners, who are relentlessly exposed to their loved one’s difficulties and may have to accommodate symptoms to a greater degree, e.g. giving reassurance, changing routines and cleaning items on behalf of the sufferer. This is likely to increase anxiety, tension and conflict in the home environment.

The changes in presentation and processes described above are important to consider in formulation and treatment (see ‘What to consider in CBT’ section below).

## CBT or not CBT? That is the question

CBT is the recommended and evidence-based psychological treatment for adults and children with OCD (National Institute for Health and Clinical Excellence, 2005). During the lockdown, services may offer treatment remotely via video-conferencing and telephone. Studies have shown this is a viable treatment option for many, and outcomes are equivalent to face-to-face CBT (e.g. Wootton, 2016). It is important to consider a range of factors before embarking on this work.

There are practical considerations such as whether someone can access remote treatment, e.g. availability of personal computers or devices, use of a quiet and confidential space for sessions and access to session materials. Clinicians have noted some advantages of remote CBT, for example people using their telephones in sessions and the ability for them to move around in areas where their OCD is triggered, making the work more ecologically valid. Some groups may struggle with remote CBT, such as young people with autism spectrum disorder (ASD) or attention deficit hyperactivity disorder (ADHD). In such cases, some services are offering face-to-face treatment in clinic with service users and therapists both wearing personal protective equipment (PPE) such as masks. PPE may impact on rapport building or undermine some in-session work, e.g. coming into contact with contaminants. Additionally, there may be travel restrictions such as not being able to use public transport, which may make accessing CBT in clinic difficult. Overall, the pros and cons of the mode of delivery (remote *vs* in clinic) need to be discussed with the client before deciding which approach to take.

Given the context of likely heightening of general anxiety and uncertainty, a discussion with the client is needed about whether they feel able to commit to therapy and try to overcome their difficulties in the context of COVID-19. It is important to consider whether commencing CBT in the current climate has the potential to increase risk (e.g. self-harm), and if so, whether a risk management plan can effectively be put in place in current circumstances, so that the individual has access to the support they need (Veale *et al*., 2009). Clinicians need also to consider whether government guidelines will restrict the feasibility of undertaking specific behavioural experiments or exposure tasks, without which treatment would be substandard. For example, a client with olfactory reference disorder needed to be able to be close to strangers in busy areas to test out their fears that others will be disgusted by their perceived smell, which is not possible to do with social distancing government guidelines, and as such treatment therefore was put on hold.

Where it is agreed that it is not the time to embark on CBT, individuals may benefit from accessing support through regular clinician contact to review and monitor how they are managing. Having said this, in our experience, the majority of people are managing to engage in CBT at this time.

## What to consider in CBT

At present there are no empirical data on the cognitive processes and behaviours that may underlie COVID-19-related OCD. Future research may reveal similarities and differences in these factors; however, our impression is that the processes are the same. Based on our early clinical observations, there are likely to be several key cognitive factors to consider in CBT. People with OCD who are high in IOU will struggle to tolerate the uncertainty associated with COVID-19, which may include not knowing whether surfaces, other people, etc. may be contaminated; whether one may have the disease oneself (and be contagious); what the appropriate level of precaution is to be taking; or whether one may have unknowingly spread the virus to others. An inflated level of responsibility for harm, where one has an exaggerated perception of one’s degree of influence over feared outcomes, will motivate excessive attempts to prevent harm. Beliefs about others (e.g. members of the public) being insufficiently careful may in turn contribute to the perception that one has to take an even greater level of precaution to compensate for others’ negligence. Closely related to inflated responsibility is the concept of ‘agency’, which is the belief that having an intrusion makes one responsible for the outcome by just having the thought (e.g. ‘having a thought about someone contracting COVID-19 makes me responsible for preventing it’). OCD sufferers’ perceptions of risk are also likely to be over-estimations and characterised by ‘all-or-nothing’ thinking, for example perceiving relatively low-risk situations (e.g. walking outside while observing social distancing guidelines) as equivalently risky to high-risk scenarios.

There are other manifestations of OCD where the nature of the fears are more qualitatively distinct from those of non-OCD sufferers, but where the content has now become focused on COVID-19. These include, for example, ‘thought–event fusion’ or magical thinking, where there is a fear that one has the power to cause COVID-19-related harm just by thinking about it (e.g. ‘if I picture a loved one contracting the virus, I will make it happen’), or ‘foretelling’, where the belief is that having an intrusion about COVID-19 represents an omen and means that it will happen in the future. Fears of ‘transfer’ may take the form of believing that having an intrusion about COVID-19 can transfer properties onto another object or person, by just having the thought (e.g. ‘if I think my hands are contaminated, then I can transfer the contamination onto objects through my thoughts’).

Treatment should begin with the development of an individualised formulation, which highlights the idiosyncratic cognitive processes and behaviours. As part of this, introducing an alternative perspective (Theory A/B) and highlighting this as a problem of excessive pre-occupations and concern about COVID-19 is important. It is likely to be important at this stage to discuss what is deemed excessive, i.e. going beyond government guidelines, significant impact on functioning, increase in distress. For clients whose OCD predated the pandemic (perhaps sub-clinically), it could be useful to consider with them whether the cognitive themes and patterns of responding may be the same. Cognitive interventions are likely to include normalisation of the uncertainty surrounding the virus and the correct levels of precaution. IOU can be introduced as a concept with reference to various metaphors, with the message that individuals in the general population will vary in their ability to tolerate the uncertainty inherent in the virus. An implication for treatment will therefore be to help increase one’s tolerance of the uncertainty. Responsibility beliefs can be evaluated using the standard methods. These include continua methods to show that responsibly behaviour in relation to the virus is not all-or-nothing but on a spectrum; adopting maximum levels of responsibility has unintended costs and reducing one’s level of responsibility does not equate with becoming negligent. Responsibility pie charts may be useful for clients whose attributions for preventing harm are excessively focused on themselves. Cost–benefit analyses will invariably form part of these conversations, drawing attention to the unintended consequences of continuing with the existing strategy. All-or-nothing styles can be addressed through continua methods and guiding the client to consider the grey area and sharing with them that we are living in this area together.

Behavioural interventions should be set up to test an emerging, alternative cognitive account of the problem (Theory B) as one of excessive worry and pre-occupation about COVID-19. Processes such as IOU, excessive responsibility, all-or-nothing thinking and over-estimation of risk may also be targeted in behavioural tasks. The underlying principles of behavioural interventions in this context will therefore be to carry out exposure to uncertainty/perceived responsibility for harm, within the bounds of the current context. The aims are to develop tolerance of uncertainty and widen the bandwidth of behaviour in line with a more functional level of responsibility.

On the surface, behavioural tasks usually associated with the treatment of OCD may appear to be contraindicated by public health advice and common-sense precaution-taking. When collaboratively developing tasks with clients, it is important to tailor tasks considering new emerging evidence about COVID-19, including information on viral loading and vulnerability groups (e.g. elderly, ethnic minorities, pregnant women). Considering environments where there may be low viral loading (e.g. open outdoor spaces) and working up to higher viral loading environments (e.g. small indoor spaces) can be one way to increase the difficulty of tasks. Most people are in the low vulnerability group, therefore tasks collaboratively developed with the client should include higher risk, e.g. go outside for a walk, touch a handrail and not handwash when they get home. If working with someone in a vulnerable group, tasks may need to be tailored to consider this, e.g. go outside for a walk, touch handrail and do a 20 second handwash when they get home.

As therapists we help our clients to weigh up their choices and to help them to choose to begin to bravely take risks which are either not present in reality or are exaggerated. This may be a more anxiety-provoking time for therapists too, as we work out our new limits in doing behavioural experiments or exposure tasks; we will have to be safe and within ethical codes, whilst also enabling our clients to take the necessary ‘risks’ to overcome their OCD. Each task would need to be developed considering the cost–benefit of task, calculating the risk (viral loading plus vulnerability factors) against the potential benefit of challenging the OCD.

Specific exposure tasks will be guided by personalised therapy goals that are meaningful to the client and enable them to live as functionally as possible during the crisis. Tasks may therefore include activities that are outside the client’s current bandwidth but which many people without OCD are still willing to engage in.

## Special groups

### Young people

There is growing awareness of the potential emotional and developmental impact of COVID-19 restrictions on children and young people (e.g. Lee, 2020), with substantial changes to their daily structure, environments and learning opportunities. For young people with neurodevelopmental conditions such as ASD (where predictability and routine may be key to emotional coping), ADHD (who are often reliant on regulation from physical activity) or where parenting capacity is reduced by other difficulties within the family, this impact may be increased. It is through this lens that formulation and interventions for young people experiencing OCD in the context of COVID-19 should be understood. Although some provision remains in place for vulnerable children and young people, many parents have reduced access to support and respite. This unfortunately has the potential for spiralling distress and family burn-out. Therapists should consider whether CBT-informed work with parents to contain and reduce their accommodation of OCD symptoms, for example, may have greater benefits in such situations than individual CBT.

As with adults, there are many young people who are able to make use of remote CBT for OCD (Lenhard *et al*., 2017; Turner *et al*., 2014). For those already familiar with online communication, the switch to this way of working may prove a smoother transition than for their adult counterparts or indeed therapists. The age and developmental profile of a young person, the availability of a quiet and calm space (alongside a parent if appropriate) in which to conduct therapy sessions, the ability to tolerate sustained screen time in a focused way and the opportunities for meaningful exposure will all influence the decision to undertake CBT.

For a young person out of school, with no direct contact with peers or indeed no time spent independently from their parents, some ingenuity may be required to identify exposure tasks that tap into the individual’s core OCD fears. This may be the time to adopt creative imagery-based tasks, to recruit grandparents and friends as online ‘stooges’ or to exploit the growing number of platforms available for exploring virtual worlds. Helpful modifications to sessions may include the use of sensory objects or materials to improve concentration and attention, drawing facilities to help them to make their ideas more concrete, use of interfaces with shared screen capabilities to enable visual aids, shorter and more frequent sessions or greater reliance on recording and recapping of session information throughout the week.

### Perinatal women

Anxiety disorders affect approximately 15% of perinatal women (Dennis *et al*., 2017), and OCD affects approximately 2–4% of women in the perinatal period, usually defined as pregnancy to 12 months postpartum (Fairbrother *et al*., 2016a). The combination of prolonged uncertainty regarding the outcome (the baby’s health and wellbeing), a myriad of potential threats and high responsibility for a vulnerable infant are particularly pertinent to the context of pregnancy and the postpartum period. There are often new incident OCD disorders during the perinatal period, in particular if there are additional risks and complications within pregnancy (Fairbrother *et al*., 2016b). The additional concern caused by the risk of contracting COVID-19 is likely to be causing perinatal women with OCD more distress and may trigger new anxiety disorders in some.

CBT is always adapted to the person’s context, with the aim of achieving behaviour relative to someone in a similar position, i.e. an expectant parent without OCD. For pregnant and postnatal women with OCD, experiments involving exposure might be less extreme than experiments outside this context, and this is further modified within the bounds of current restrictions, for example additional caution in late pregnancy. Intrusive thoughts of deliberately harming the infant are very common in perinatal women and distress may be exacerbated by the constant close proximity and lack of normative information available, intensified by lockdown. Gaining a sense of the range of normal parenting and infant behaviour is key to working with perinatal OCD, and encouraging women to access general online peer support and antenatal groups is an important part of treatment during social distancing. It may also help with symptoms of low mood which are a likely consequence of current limitations, and often play a role in maintaining perinatal OCD (Challacombe *et al*., 2016). Additionally, the need to visit hospitals for some (albeit pared back) antenatal appointments as well as to give birth in hospital is a source of understandable worry, and women may need support in thinking through the best ways to manage these situations and problem solving any issues that may present in the context of their particular fears.

## What happens when it is all over?

It might be predicted that a return to more normal levels of activity, and a decrease in the need to adopt such stringent and hypervigilant efforts to fight COVID-19 will be celebrated by the public generally. However, it seems almost certain that we will be doing so in an atmosphere of ongoing uncertainty, at least until a vaccine is developed. We are going to have to learn to live with the virus, and some people may find it hard to leave the ‘enhanced/emergency COVID-19 rules’ behind. There is likely to be a general level of anxiety, and it is probably true that we are all going to have to take ‘calculated risks’ in order to regain a more fulfilling and enjoyable way of life. There may have to be cost–benefit analyses for us all as we calculate the level of risk we are individually prepared to take in order to get our lives back to our ‘new normal’. Some people, perhaps those with co-morbid health problems, may choose or be advised to remain living in a fairly restricted way, as the risk for them will be much higher. Others will happily adopt a much more relaxed approach.

There is nothing at this point to say there will be an increase in rates of OCD in the general population. However, it may be that this level of threat has been traumatic for many people, and this may be an opportunity to pick up those people who are at risk of further exacerbation of anxiety and worsening of symptoms over time. People do not usually present with OCD until an average 6 years after onset (Stobie *et al*., 2007), despite OCD symptom severity increasing over this time. This could be a good opportunity to help this population identify early warning signs of OCD, such as excessive fear and pre-occupation with COVID-19, inability to resume regular activities or inability to drop safety behaviours that were functional during the pandemic, when the advice is that these are no longer necessary. Awareness and knowledge of OCD and its development have generally increased, and it might be hoped that we can use COVID-19 as an opportunity to educate people to identify symptoms at an early stage and seek help.

## Conclusion

People with OCD are likely to be more susceptible to the mental health impact of COVID-19. The presentation of OCD in the context of the pandemic, in terms of the cognitive and behavioural processes, are likely to be broadly similar to presentations before the pandemic, but the content of the fear will have changed. Government guidance on COVID-19 will have an impact on the delivery of CBT; however, in our experience effective treatment can still be carried out for a majority of people. When therapists and clients are developing behavioural experiments and exposure tasks which involve contact with potential COVID-19 contaminants, they need to consider the current understanding of COVID-19, such as viral loading and individual vulnerability factors. The costs and benefits of such tasks need to be weighed up with regard to the risk of contracting COVID-19 against the benefits of overcoming OCD. Whilst there is no evidence to suggest there will be an increase in OCD in the aftermath of the pandemic, there still may be opportunities to help this population identify early warning signs of OCD and access support sooner.

## Key practice points

(1) COVID-19 may change the content of the threat for those with OCD, but the cognitive and behavioural processes involved are similar.
(2) Current government guidance on managing COVID-19 may make aspects of treatment delivery and content more difficult, but for the vast majority of people with OCD, CBT can be delivered effectively.
(3) When undertaking CBT for OCD, attention must be given to how best to set up behavioural experiments and exposure tasks in light of current understanding of COVID-19, i.e. viral loading and individual vulnerability factors.
(4) Therapists are working within new limits in doing CBT for OCD, and have to balance the needs to be safe and work within ethical codes, with enabling clients to take the necessary risks to overcome their OCD.
